# Non-effectiveness of Ivermectin on Inpatients and Outpatients With COVID-19; Results of Two Randomized, Double-Blinded, Placebo-Controlled Clinical Trials

**DOI:** 10.3389/fmed.2022.919708

**Published:** 2022-06-16

**Authors:** Mohammad Sadegh Rezai, Fatemeh Ahangarkani, Andrew Hill, Leah Ellis, Manya Mirchandani, Alireza Davoudi, Gohar Eslami, Fatemeh Roozbeh, Farhang Babamahmoodi, Nima Rouhani, Ahmad Alikhani, Narges Najafi, Roya Ghasemian, Hossein Mehravaran, Azin Hajialibeig, Mohammad Reza Navaeifar, Leila Shahbaznejad, Golnar Rahimzadeh, Majid Saeedi, Reza Alizadeh-Navai, Mahmood Moosazadeh, Shahab Saeedi, Seyedeh-Kiana Razavi-Amoli, Shaghayegh Rezai, Fereshteh Rostami-Maskopaee, Fatemeh Hosseinzadeh, Faezeh Sadat Movahedi, John S. Markowitz, Reza Valadan

**Affiliations:** ^1^Pediatric Infectious Diseases Research Center, Communicable Diseases Institute, Mazandaran University of Medical Sciences, Sari, Iran; ^2^Antimicrobial Resistance Research Center, Communicable Diseases Institute, Mazandaran University of Medical Sciences, Sari, Iran; ^3^Department of Pharmacology and Therapeutics, Liverpool University, Liverpool, United Kingdom; ^4^Faculty of Medicine, School of Public Health, Imperial College London, London, United Kingdom; ^5^Department of Clinical Pharmacy, Faculty of Pharmacy, Cardiovascular Research Center, Mazandaran University of Medical Sciences, Sari, Iran; ^6^Gastrointestinal Cancer Research Center, Non-communicable Diseases Institute, Mazandaran University of Medical Sciences, Sari, Iran; ^7^Department of Internal Medicine, Pulmonary and Critical Care Division, School of Medicine, Mazandaran University of Medical Sciences, Sari, Iran; ^8^Department of Pharmaceutics, Faculty of Pharmacy, Mazandaran University of Medical Sciences, Sari, Iran; ^9^Student Research Committee, Mazandaran University of Medical Sciences, Sari, Iran; ^10^Department of Microbiology and Virology, Mashhad University of Medical Sciences, Mashhad, Iran; ^11^Department of Pharmacotherapy and Translational Research, Center for Pharmacogenomics and Precision Medicine, University of Florida, Gainesville, FL, United States; ^12^Department of Immunology and Molecular and Cell Biology Research Center, Faculty of Medicine, Mazandaran University of Medical Sciences, Sari, Iran

**Keywords:** ivermectin, COVID-19, inpatients, outpatients, effectiveness

## Abstract

**Background:**

Ivermectin which was widely considered as a potential treatment for COVID-19, showed uncertain clinical benefit in many clinical trials. Performing large-scale clinical trials to evaluate the effectiveness of this drug in the midst of the pandemic, while difficult, has been urgently needed.

**Methods:**

We performed two large multicenter randomized, double-blind, placebo-controlled clinical trials evaluating the effectiveness of ivermectin in treating inpatients and outpatients with COVID-19 infection. The intervention group received ivermectin, 0.4mg/kg of body weight per day for 3 days. In the control group, placebo tablets were used for 3 days.

**Results:**

Data for 609 inpatients and 549 outpatients were analyzed. In hospitalized patients, complete recovery was significantly higher in the ivermectin group (37%) compared to placebo group (28%; RR, 1.32 [95% CI, 1.04–1.66]; *p*-value = 0.02). On the other hand, the length of hospital stay was significantly longer in the ivermectin group with a mean of 7.98 ± 4.4 days compared to the placebo receiving group with a mean of 7.16 ± 3.2 days (RR, 0.80 [95% CI, 0.15–1.45]; *p*-value = 0.02). In outpatients, the mean duration of fever was significantly shorter (2.02 ± 0.11 days) in the ivermectin group versus (2.41 ± 0.13 days) placebo group with *p* value = 0.020. On the day seventh of treatment, fever (*p*-value = 0.040), cough (*p*-value = 0.019), and weakness (*p*-value = 0.002) were significantly higher in the placebo group compared to the ivermectin group. Among all outpatients, 7% in ivermectin group and 5% in placebo group needed to be hospitalized (RR, 1.36 [95% CI, 0.65–2.84]; *p*-value = 0.41). Also, the result of RT-PCR on day five after treatment was negative for 26% of patients in the ivermectin group versus 32% in the placebo group (RR, 0.81 [95% CI, 0.60–1.09]; *p*-value = 0.16).

**Conclusion:**

Our data showed, ivermectin, compared with placebo, did not have a significant potential effect on clinical improvement, reduced admission in ICU, need for invasive ventilation, and death in hospitalized patients; likewise, no evidence was found to support the prescription of ivermectin on recovery, reduced hospitalization and increased negative RT-PCR assay for SARS-CoV-2 5 days after treatment in outpatients. Our findings do not support the use of ivermectin to treat mild to severe forms of COVID-19.

**Clinical Trial Registration:**

www.irct.ir IRCT20111224008507N5 and IRCT20111224008507N4.

## Introduction

Severe acute respiratory syndrome coronavirus-2 (SARS-CoV-2) is a novel coronavirus responsible for the pandemic which initiated in 2019 and persists today (1, 2). While the rapid development of vaccines against coronavirus disease-2019 (COVID-19) is a striking ongoing process, significant parts of the world population remain at risk for this infection (1). On the other hand, development of a new COVID-19 variant should always be anticipated. Currently, there is no precise, effective medication for COVID-19 (3). Achieving an effective, safe, easy-to-administer, and low-cost treatment for inpatients or outpatients is urgently needed, particularly in low-income countries where the availability of COVID-19 vaccines is inaccessible or slow (4, 5). Out of hundreds of drugs that have been used for the treatment of COVID-19, mainly for hospitalized patients, just a few of them are adequate or received a conditional marketing authorization (6–9). Moreover, numerous clinical trials to assess the potential of existing licensed drugs with well-established safety profiles have been conducted or are underway on COVID-19 patients to accelerate the identification of appropriate medication for treatment or prevention of infection. In preclinical studies, various repurposed drugs have been demonstrated as potential inhibitors of one or more steps of the SARS-CoV-2 lifecycle (6–8, 10–12). However, evidence from primary studies is inadequate, and more recent reports from large-scale clinical trials are needed. Nevertheless, clinical trials for repurposed medications for inpatients with COVID-19 are ongoing (6, 10, 11, 13–15). Ivermectin is a semisynthetic FDA-approved broad-spectrum anti-parasitic drug. In addition to anti-parasitic effects such as its therapeutic role in onchocerciasis and strongyloidiasis, this drug offered new clinical applications due to its ability to be repurposed to treat new classes of diseases (16–19). With *in vitro* antiviral activity against the SARS-CoV-2 virus, ivermectin has been introduced as a promising therapeutic candidate for COVID-19. In a Vero-hSLAM cell culture model, a single dose of ivermectin stimulated about a 5000-fold decrease in the viral RNA of SARS-CoV-2 at 48 h (20).

Additionally, ivermectin was shown to manage the infections caused by various RNA viruses such as influenza, respiratory syncytial virus, dengue, and rabies virus (20). Ivermectin demonstrates immunomodulatory and anti-inflammatory effects in preclinical models by suppressing the production of inflammatory mediators. Moreover, it can promote human immunity by enhancing the IL-1 production and additional cytokines, stimulating superoxide anion increasing, and improving the lymphocyte response to mitogens (20–23). Moreover half-life of ivermectin in humans is long (12–36 h), whereas metabolites could persist for up to a few days (24). Since the beginning of the COVID-19 pandemic, many observational and clinical trials studies with various doses and schedules have evaluated ivermectin as a treatment or a prophylaxis option for COVID-19. Although many studies have shown favorable effects of ivermectin in the treatment COVID-19, the results are not uniform, and sometimes contradictory (25–28). In general, most of these studies do not have a sufficient sample size or have methodological limitations. The current studies have been inconclusive largely due to the lack of clinically essential data, such as reducing mortality, length of hospital stay, need for invasive mechanical ventilation, and decreased time to clinical improvement in COVID-19 patients. The World Health Organization exclusively allowed for this drug in clinical trials for COVID-19 patients. Since the evidence to support the use of ivermectin as a treatment or prophylaxis option for COVID-19 infection is conflicting, herein, we describe two multicenter studies assessing the effectiveness of ivermectin on inpatients and outpatients with COVID-19 in two double-blind randomized placebo-controlled clinical trials.

## Materials and Methods

### Trials Design

These two separated multicenter randomized, double-blind, placebo-controlled clinical trial studies were performed to evaluate the effectiveness of ivermectin in treating inpatients and outpatients with COVID-19 infection. The diagnosis of COVID-19 was confirmed with the following criteria; positive result from real-time reverse transcriptase-PCR (RT-PCR) assay for severe acute respiratory syndrome coronavirus-2 (SARS-CoV-2) using nasopharyngeal swab; direct detection of SARS-CoV-2 viral proteins (antigens) in nasal swabs and other respiratory secretions using lateral flow immunoassays (rapid test); abnormalities on chest computed tomography (CT) compatible with COVID-19 (ground-glass opacity, halo sign, reversed halo sign, and patchy infiltration). The drugs were discontinued if the patient developed any serious side effects. All of the participants received appropriate antibiotics or supplemental oxygen as indicated.

### Trial of Inpatients

#### Participants in Inpatients Trial

Recruitment began in February 2021 and ended in August 2021. There were six cities for trial sites in Mazandaran province, including Sari (Boali and Imam hospitals), Qaemshahr (Razi hospital), Neka (Imam Hossein hospital), Behshar (Imam hospital), Ramsar (Imam Sajjad hospital), and Amol (Imam hospital). The selection criteria to select inpatient which the protocol was published at https://www.irct.ir/trial/54402 were included patients with moderate COVID-19 [clinical signs of pneumonia (fever, cough, dyspnea, and tachypnea)] to severe COVID-19 (with clinical signs of pneumonia plus one of the following: respiratory rate >30 breaths/min; severe respiratory distress; or SpO2 <90% on room air), patients ≥18 years old and weight ≥15 kg with the ability to provide informed consent. Also patients were excluded if they were unable to take oral medication, had a known history of ivermectin allergy, were pregnant or breastfeeding, had a history of chronic liver or renal disease; received treatment with warfarin, an angiotensin-converting enzyme inhibitor, or an angiotensin II receptor antagonist; or had acquired immunodeficiency.

#### Randomization and Masking of Inpatients Trial

Random allocation was done by the study methodologist using a random number generator in R (4.0.4 version). Randomization occurred at Bu-Ali Sina hospital. A table of random numbers from 1 to 891 was prepared in a non-sequential and scattered manner, and the numbers were assigned to two intervention and control groups of 447 and 444 cases, respectively. Participants and clinicians were masked to the randomization process and group allocation. In this study, the research pharmacists were unmasked and responsible for the preparation and distribution of all interventions. Participants who met eligibility criteria were randomly assigned in a 1:1 ratio to receive either ivermectin plus the national standard care (SOC) or placebo plus the SOC on day one.

#### Interventions on Inpatients Trial

The intervention group received a single oral dose (0.4 mg/kg) of ivermectin per day for 3 days utilizing 6-mg tablets (Alborz Daru Company, Tehran, Iran), at the following rounded off weight-based doses: 15–30 kg, 6 mg; 31–45 kg, 12 mg; 46–60 kg, 18 mg; 61–75 kg, 24 mg; and > 75 kg, 30 mg for 3 days. Also, in the control group, placebo tablets (Alborz Daru Company, Iran) with the similar appearance, taste, smell, shape, color, and weight-based dose of ivermectin were used for 3 days. The bottles of ivermectin and placebo were identical.

#### Trial Procedures on Inpatients

All participants who entered the trial underwent detailed characterizations, including Socio-demographical features (age, sex, body mass index [BMI], living area, and level of education) and preexisting comorbidities. A physical examination was conducted (including respiratory rate, blood oxygen saturation, and chest auscultation) on the first day of admission. Also, clinical evaluation (symptoms, vital signs, the severity of infection, and medications and adverse events of ivermectin) were collected. All data were recorded once daily in the checklist. Moreover, all the pages in the patients’ files were photographed on the discharge day from the hospital. The compliance of the data checklist and the items in the participants’ files was checked.

#### Outcomes of Inpatient Trial

The primary outcome measure was a clinical improvement, including; resolution of main symptoms within the hospital admission period, including tachypnea, dry cough, and increasing oxygen saturation by day 7; recovery including complete recovery (resolving main complaints by discharge day) and relative recovery (remaining main complaints at discharge day) and; progression (deterioration of symptoms). Secondary outcomes included length of hospital stay, intensive care unit (ICU) admission, need for an invasive and non-invasive ventilator, drug-induced adverse events, and death.

### Trial in Outpatients

#### Participants in Outpatient’s Trial

Recruitment began in February 2021 and ended in August 2021. The selection criteria to select outpatient which the protocol was published at https://www.irct.ir/trial/53949 were.

COVID-19 patients referred to the family physician, infectious disease specialist, pediatrician, or pediatric infectious disease subspecialist in outpatient clinics were considered. Inclusion criteria included; patients with positive diagnostic by RT-PCR assay for SARS-CoV-2 using a nasopharyngeal swab ≤ 4 days prior to screening or positive rapid COVID-19 test, without evidence of viral pneumonia or hypoxia, with age more than 5 years old, weight more than 15 kg and able to take oral medication. Also exclusion criteria included; unable to take oral medication or sign the informed consent, patients who took antiviral before or during the study, known history of ivermectin allergy, pregnancy or breastfeeding, a history of chronic liver and/or renal disease; receipt of treatment with warfarin, an angiotensin-converting enzyme inhibitor, or an angiotensin II receptor antagonist; and acquired immunodeficiency.

#### Randomization and Masking in Outpatient’s Trial

Participants who met eligibility criteria were randomized in a 1:1 ratio to receive either ivermectin plus the SOC or a placebo plus the SOC on day one. Randomization was done by the study methodologist using a random number generator with R (4.0.4 version). A table of random numbers from 1 to 582 was prepared in a non-sequential and scattered manner, and the numbers were assigned to two intervention (282 cases) and control groups (300 cases). Participants and clinicians were masked to the randomization process or group allocation. In this study, the pharmacist was unmasked and responsible for the preparation and distribution of all interventions.

#### Interventions on Outpatients

The intervention group received 0.4 mg/kg of body weight per day for 3 days of ivermectin utilizing 6-mg oral tablets (made by Alborz Daru Company, Tehran, Iran), plus the SOC. Also the control group received a placebo (0.4 mg/kg/day for 3 days; made by Alborz Daru Company, Iran), plus the SOC. Ivemectin and placebo tablets were similar in appearance, taste, smell, shape and color, and weight-based dose.

#### Trial Procedures of Outpatients

Patients were recruited by referral from a family physician or infectious disease specialist and underwent a medical screening visit before randomization. All participants who entered the trial underwent detailed characterizations, including demographical features, physical examination, clinical evaluation including symptoms, vital signs, preexisting comorbidities, and medications specified for COVID-19, and adverse events of ivermectin from the first visit day to day seven. Also, a trained nurse collected nasopharyngeal swabs from all potential patients on day five after treatment for RT-PCR assay for SARS-CoV-2. All data were recorded in the checklist.

#### Outcomes of Outpatient’s Trial

The primary outcome measure was the time to resolution of symptoms, recovery including complete recovery (resolving main complaints at the sixth day) and relative recovery (remaining main complaints at sixth day); progression (needing hospitalization) and negative RT−PCR result at 5 days. Secondary outcomes included need ICU admission, drug-induced adverse events, and death.

### Statistical Analysis

Data were analyzed using IBM SPSS Statistics version 20.0 (IBM, Armonk, NY, United States). In the time of study design, several clinical trials were ongoing from 45 to 600 sample sizes. To attain a statistical power of at least 0.95 with an alpha error of 0.05, and more than previous studies populations the sample size up to 1000 patients were calculated. Means (SD) were used for reporting quantitative data, and frequency and percentage, for qualitative variables. For comparison of differences between intervention and control group, *t*-test and χ^2^ tests were used. The Kaplan–Meier Breslow method was used for estimating the duration of hospitalization and symptoms in both groups. A *p-*value of < 0.05 was considered statistically significant. The relative risk (RR) of symptoms on the seventh day of following in the ivermectin and placebo groups was calculated with 95% confidence intervals (95%CI); relative confidence intervals not including 1 were considered statistically significant.

## Results

### Inpatients Characteristics

**FIGURE 1:**
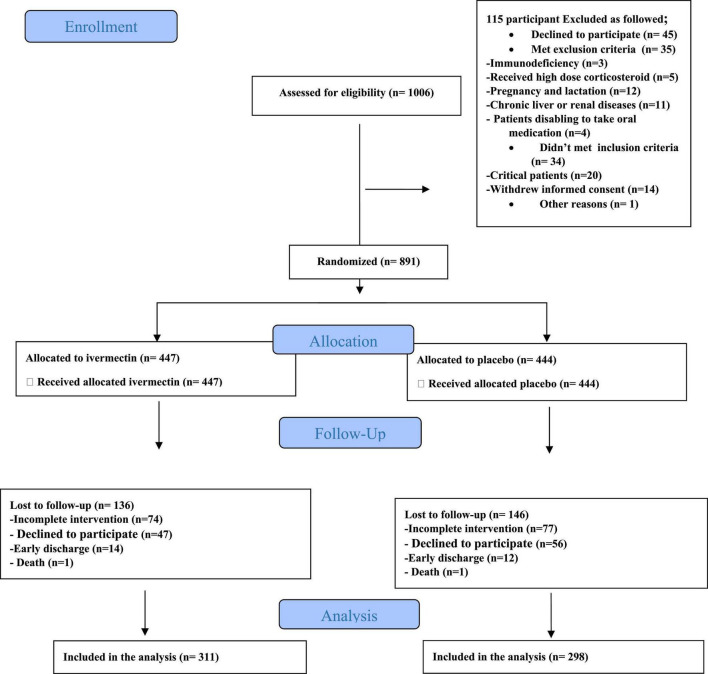
Enrollment, allocation, follow up and assignment of inpatients.

In total, 1006 participants with COVID-19 were screened for eligibility between February 19, 2021, and August 14, 2021. Out of these participants, 115 were excluded. The main reasons for their exclusion were declining to participate (*n* = 45); met with exclusion criteria including immunodeficiency (*n* = 3), receiving high dose corticosteroid (*n* = 5), pregnancy or lactation (*n* = 12), and chronic liver or renal diseases (*n* = 11) and unable to take oral medication (*n* = 4); participants who did not meet inclusion criteria including, critical patients (*n* = 20), participants who withdrew informed consent (*n* = 14); and other reasons (*n* = 1). Finally, 891 eligible participants were enrolled and included in the trial and randomized, of which 282 patients were lost to follow-up (136 individuals in the ivermectin group and 146 in the placebo group). The main reasons for the loss in follow-up were incomplete intervention (*n* = 151), declined to be a participant (*n* = 103), early discharge (*n* = 26), and death (*n* = 2). Eventually, 609 patients (311 individuals in the ivermectin arm and 298 in the control arm) completed 7 days of follow-up (Figure 1). Participants in both arms had generally well-balanced baseline characteristics (Table 1). The socio-demographics and baseline characteristics of inpatients are shown in Table 1. The majority of enrollments, 318 (52.2%), were female. The mean ± standard deviation of participants’ age was 53.79 ± 15.3 (range 23–96). 73.32% of participants had a body mass index (BMI) of more than 30 kg/m^2^. Also 72.5% of participants lived in urban regions. The proportion of patients with moderate illness was 50.8% and 57% versus severe cases was 49.2% and 43% in the ivermectin and placebo arms, respectively, with no statistically significant difference between the two groups. Totally 345 (56.7%) patients had at least one comorbidity. The most common underlying illnesses were diabetes 31.7%, hypertension 28.4%, and cardiovascular disease 12.2%. Participants’ symptoms on the first day of admission are shown in Table 2. The most common symptoms were dyspnea 67.5%, dry cough 61.7%, and fever 59.6%. Moreover, the mean duration of symptoms before randomization was not significantly different in both groups (7.36 ± 3.43 days in the ivermectin group and 6.98 ± 3.63 days in the placebo group) with a *p*-value = 0.309. There were no significant differences in the socio-demographic, baseline characteristics, comorbidities, and presenting symptoms in the two arms (Tables 1, 2). Participants were recruited from seven hospitals to both arms in a balanced manner (*p* = 0.858). Participants were hospitalized on similar dates in the two treatment arms (*p* = 0.38). The list of concomitant medications on enrollment is described in Table 3. The most common concomitant medications in patients were antiviral (remdesivir for 98.2% of patients), glucocorticoid (dexamethasone for 90.7%) and anticoagulant (heparin and enoxaparin for 85.1% of patients). Overall, concomitant medications were balanced across the two arms and there were no significant differences in concomitant medications administrated in both groups.

**TABLE 1:** The socio-demographics and baseline characteristics of inpatients.

Socio-demographics and baseline characteristics		Total (*n* = 609)	Ivermectin (*n* = 311)	Placebo (*n* = 298)	*P-value*
Age	Mean (range)	53.79 (23–96)	53 (23–95)	54 (25–96)	0.333
Gender	Male	291 (47.8%)	151 (48.6%)	140 (47.0%)	0.698
	Female	318 (52.2%)	160 (51.4%)	158 (53.0%)	
Obesity	BMI*≥30	404/551 (73.32)	205/274 (74.82)	199/277 (71.84)	0.430
	BMI≤30	147/551 (26.88)	69/274 (25.18)	78/277 (28.16)	
Living place	Urban	428 (72.5%)	222 (74.2%)	206 (70.8%)	0.347
	Rural	162 (27.5%)	77 (25.8%)	85 (29.2%)	
Education	<Bachelor’s degree	395 (79.5%)	196 (76.9%)	199 (82.2%)	0.139
	≥Bachelor’s degree	102 (20.5%)	59 (23.1%)	43 (17.8%)	
Contact tracing of COVID-19	Contact history of the suspect	138 (22.7%)	78 (25.1%)	60 (20.1)	0.086
	Definitive positive contact history	99 (16.3%)	59 (19.0%)	40 (13.4%)	0.128
	Recent travel history	15 (2.5%)	9 (2.9%)	6 (2.0%)	0.483
Duration of symptom before randomize (day)	Mean ± standard deviation	7.18 ± 3.52	7.36 ± 3.43	6.98 ± 3.63	0.309
Oxygen saturation	Mean ± standard deviation	92.49 ± 5.42	92.104 ± 5.26	92.89 ± 5.89	0.110
Severity of Disease	Severe	281 (46.1%)	153 (49.2%)	128 (43.0%)	0.072
	Moderate	328 (53.9%)	158 (50.8%)	170 (57%)	
Comorbidities	At least one comorbidity *n* (%)	345 (56.7%)	175 (56%)	170 (57%)	0.911
	Diabetes	193 (31.7%)	103 (33.1%)	90 (30.2%)	0.439
	Hypertension	173 (28.4%)	85 (27.3%)	88 (29.5%)	0.608
	Cardiovascular disorders	74 (12.2%)	38 (12.2%)	36 (12.1%)	0.958
	Dyslipidemia	54 (8.9%)	29 (9.3%)	25 (8.4%)	0.685
	Hypothyroidism	51 (8.4%)	30 (9.6%)	21 (7.0%)	0.247
	Asthma	18 (3.0%)	11 (3.5%)	7 (2.3%)	0.531

*BMI, body mass index.

**TABLE 2:** Symptoms of inpatients in the first day of admission.

Symptoms	Total (*n* = 609)	Ivermectin (*n* = 311)	Placebo (*n* = 298)	*P-value*
Dyspnea	411 (67.5%)	209 (67.2%)	202 (67.8%)	0.878
Dry cough	376 (61.7%)	198 (63.7%)	178 (59.7%)	0.318
Fever	363 (59.6%)	174 (55.95)	189 (63.4%)	0.060
Weakness	202 (33.2%)	106 (34.1%)	96 (32.2%)	0.624
Body pain	195 (32.0%)	103 (33.1%)	92 (30.9%)	0.553
Chills	194 (31.9%)	91 (29.3%)	103 (34.6%)	0.160
Anorexia	190 (31.2%)	108 (34.7%)	82 (27.5%)	0.055
Nausea	153 (25.1%)	78 (25.1%)	75 (25.2%)	0.980
Headache	128 (21.0%)	67 (21.5%)	61 (20.5%)	0.745
Vomiting	83 (13.6%)	44 (14.1%)	39 (13.1%)	0.703
Vertigo	58 (9.5%)	31 (10.0%)	27 (9.1%)	0.703
Sore throat	52 (8.5%)	32 (10.3%)	20 (6.7%)	0.114
Sputum cough	50 (8.2%)	28 (9.0%)	22 (7.4%)	0.466
Diarrhea	53 (8.7%)	29 (9.3%)	24 (8.1%)	0.578
Abdominal pain	48 (7.9%)	28 (9.0%)	20 (6.7%)	0.294
Insomnia	24 (3.9%)	16 (5.1%)	8 (2.7%)	0.119
Arthralgia	19 (3.1%)	11 (3.5%)	8 (2.7%)	0.545
Anosmia	18 (3.0%)	13 (4.2%)	5 (1.7%)	0.068
Tachypnea	9 (1.5%)	5 (1.6%)	4 (1.3%)	1.000

**TABLE 3:** List of concomitant medications, vitamins and minerals supplements prescribed for inpatients.

	Medication	Total (*n* = 609)	Ivermectin (*n* = 311)	Placebo (*n* = 298)	*P-value*
Antiviral	Remdesivir	598 (98.2%)	307 (98.7%)	291 (97.7%)	0.325
	Hydroxychloroquine	213 (35.0%)	100 (32.2%)	113 (37.9%)	0.136
	Favipiravir	4 (0.7%)	1 (0.3%)	3 (1.0%)	0.363
Antibiotics	Doxycycline	285 (48.4%)	139/301 (46.2%)	146/288 (50.7%)	0.273
	Ceftriaxone	244 (41.4%)	122 (40.5%)	122 (42.4%)	0.652
	Clindamycin	52 (8.8%)	22 (7.3%)	30 (10.4%)	0.184
	Vancomycin	35 (5.9%)	13 (4.3%)	22 (7.6%)	0.088
	Imipenem	32 (5.4%)	14 (4.7%)	18 (6.3%)	0.392
	Meropenem	20 (3.43%)	6 (2.0%)	14 (4.9%)	0.055
	Azithromycin	20 (3.43%)	9 (3.0%)	11 (3.8%)	0.579
	Levofloxacin	5 (0.8%)	3 (1.0%)	2 (0.7%)	1.000
	Ciprofloxacin	5 (0.8%)	2 (0.7%)	3 (1.0%)	0.680
Glucocorticoid	Dexamethasone	342 (90.7%)	165 (91.7%)	177 (89.8%)	0.543
	Methylprednisolone	154 (43.1%)	77 (45.3%)	77 (41.2%)	0.433
Anticoagulant	Heparin and Enoxaparin	407 (85.1%)	211 (89.0%)	196 (81.3%)	0.180
Non-steroidal anti-inflammatory drug	Naproxen	245 (53.5%)	121 (53.3%)	124 (53.7%)	0.936
	Aspirin	102 (22.3%)	49 (21.6%)	53 (22.9%)	0.727
Anti-diabetic	Insulin	117 (25.5%)	61 (26.9%)	56 (24.2%)	0.519
	Metformin	67 (14.6%)	33 (14.5%)	34 (14.7%)	0.956
Biological response modifiers	Interferon	224 (48.9%)	104/227 (45.8%)	120/231 (51.9%)	0.189
Other drugs	Famotidine	357 (74.7%)	172 (72.6%)	185 (76.8%)	0.292
	Vasopressin	27 (4.6%)	11 (3.7%)	16 (5.6%)	0.270
Vitamins and minerals supplements	VIT C	242 (52.8%)	127 (55.9%)	115 (49.8%)	0.186
	Zink	136 (28.5%)	64 (27.0%)	72 (29.9%)	0.487
	VIT D	109 (22.8%)	51 (21.5%)	58 (24.1%)	0.507

### Primary Outcomes in Inpatients

**FIGURE 2:**
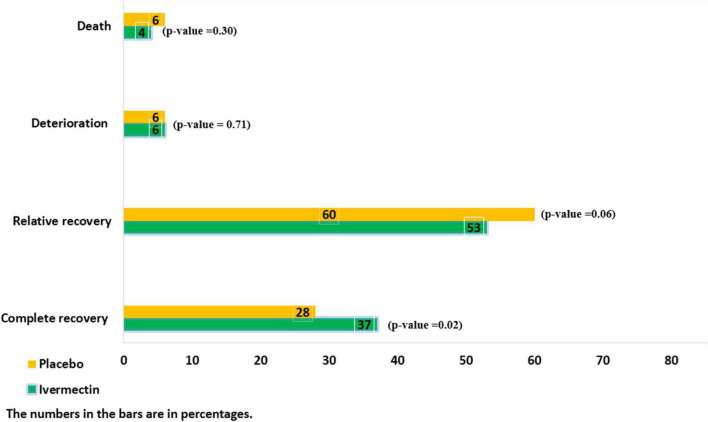
The main outcomes in inpatients with COVID-19.

There were no significant differences between the two treatment groups regarding the main symptoms, such as the persistent dry cough (until the seventh day), which was observed in 5/145 (3%)of patients in ivermectin versus 10/105 (9%) in placebo groups (RR, 0.36 [95% CI, 0.13–1.03]; *p*-value = 0.06) and tachypnea until the seventh day was absent in all participants in the ivermectin group and present in 1 participant in the control group (RR, 0.24[95% CI, 0.01–5.88] *p*-value = 0.38). The mean oxygen saturation at day 7 was 92.01 (Range: 72–99) in the ivermectin arm and 93 (Range: 48–99) in the control arm. There was no significant difference between the treatment arms (RR, –0.99 [95% CI, –2.89 to 0.91] *p*-value = 0.31). However, oxygen saturation was evaluated for only 102 participants in the ivermectin group and 95 in the control group. Complete recovery was significantly higher in ivermectin group (37%) compare to placebo group (28%; RR, 1.32 [95% CI, 1.04–1.66]; *p*-value = 0.02). The relative recovery was achieved in 60% of patients in the placebo group compared to 53% in the ivermectin group, although the differences were not significant (RR, 0.87 [95% CI, 0.76–1.00]; *p*-value = 0.06). Few patients had deterioration of symptoms, and there was no significant difference between the two treatment groups (20/311 (6%) in the ivermectin group and 17/298 (6%) in the placebo group (RR, 1.13 [95% CI, 0.60–2.11] *p*-value = 0.71; Figure 2).

### Secondary Outcomes in Inpatients

The average hospitalization stay of patients were 7.56 ± 3.8 (range 3–36) days. The length of hospital stay was significantly longer in the ivermectin group with mean 7.98 ± 4.4 days in comparison to the placebo receiving group with mean 7.16 ± 3.2 days (RR, 0.80 [95% CI, 0.15–1.45]; *p*-value = 0.02). Overall 28 patients (9%) in ivermectin group and 32 patients (11%) in placebo group were admitted to the ICU (RR, 0.84 [95% CI, 0.52–1.36]; *p*-value = 0.47). Invasive mechanical ventilator was utilized for 3% in ivermectin and 6% in placebo group (RR, 0.50 [95% CI, 0.24 –1.07]; *p*-value = 0.07). Also 244 patients (78%) in the ivermectin group and 252 patients (85%) in the control group required supplemental oxygen by non-invasive ventilation (RR, 0.93 [95% CI, 0.86–1.00]; *p*-value = 0.05). There were 13 (4%) deaths in the ivermectin arm and 18 (6%) in the placebo arm with no significant difference between the arms (RR, 0.69 [95% CI, 0.35–1.39]; *p*-value = 0.30). The drug-induced adverse events were not observed in both groups.

### Outpatient’s Results

#### Outpatient’s Characteristics

**FIGURE 3:**
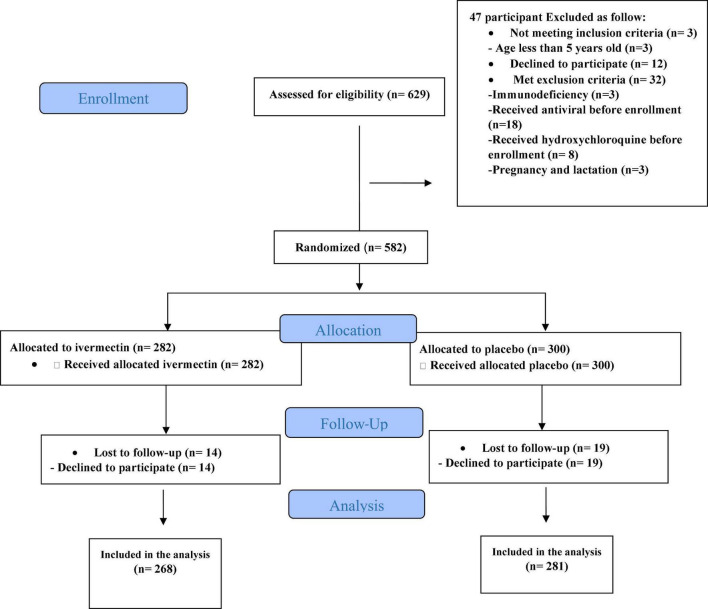
Enrollment, allocation, follow up and assignment of outpatients.

Between February 19 and August 30, 2021, of 629 SARS-CoV2 positive cases who consented were assessed for eligibility, 47 were excluded. The main reasons for their exclusion were; met with exclusion criteria including received antiviral before enrollment (*n* = 18), received hydroxychloroquine before enrollment (*n* = 8), pregnancy or lactation (*n* = 3) and immunodeficiency (*n* = 3); declining to participate (*n* = 12); participants who did not meet inclusion criteria including, age less than 5 years old (*n* = 3). Finally, 582 eligible participants were enrolled and included in the trial and randomized, of which 33 patients lost to follow-up (14 individuals in the ivermectin group and 19 in the placebo group). The reason for the loss in follow-up was due to declining to be a participant. Eventually, data for 549 patients (268 individuals in the ivermectin arm and 281 in the control arm) were analyzed (Figure 3). The participant’s mean age was 35.46 ± 17.48 years old with a range (5–87) years old. The majority of enrollments, 288 (52.46%), were male. A total of 101 (21.22%) participants had a BMI of more than 30 kg/m^2^. 74.13% of participants lived in urban regions and 112 (20.4%) participants had at least one underlying disease. The most common underlying illnesses were hypertension (7.83%) and diabetes (7.29%). Before randomization, the mean duration of symptoms was 2.99 ± 2.63 days in the ivermectin group versus 3.14 ± 3.02 days in the placebo group, which was not significantly different with a *p*- value = 0.559. The most common symptoms at the first visit were fever (52.64%), body pain (46.27%), and cough (45.36%). The socio-demographic, baseline characteristics, comorbidities, and presenting symptoms in the two arms were well balanced (Tables 4, 5). The concomitant medications are described in Table 6. The most common concomitant medication in patients were non-steroidal anti-inflammatory drugs (naproxen for 32.46% of patients), antibiotics (Azithromycin for 28.32% of patients), and vitamins or mineral supplements (vitamin C for 50.12% and Zink for 57.28% of patients). There were no significant differences in concomitant medications administrated in both groups.

**TABLE 4:** The socio-demographics and baseline characteristics of outpatients.

Socio-demographics and baseline characteristics		Total (*n* = 549)	Ivermectin (*n* = 268)	Placebo (*n* = 281)	*P-value*
Age	Mean ± standard deviation (range) year	35.46 ± 17.48 (5–87)	34.42 ± 17.72 (5–87)	36.46 ± 17.45 (5–76)	0.120
Gender	Male	288 (52.46)	140 (52.24)	148 (52.67)	0.920
	Female	261 (47.54)	128 (47.76)	133 (47.33)	
Obesity	BMI ≥ 30	101 (21.22)	52 (22.22)	193 (79.75)	0.598
	BMI ≤ 30	375 (78.78)	182 (77.78)	49 (20.25)	
Living Place	Urban	407 (74.13)	209 (77.99)	198 (70.46)	0.044
	Rural	142 (25.87)	59 (22.01)	83 (29.54)	
Education	<Bachelor’s degree	383 (70.53)	184 (69.70)	199 (71.33)	0.896
	≥Bachelor’s degree	135 (24.86)	68 (25.76)	67 (24.01)	
	Definitive positive contact history	226 (41.17)	110 (41.04)	116 (41.28)	0.671
	Contact history of the suspect	183 (33.33)	86 (32.09)	97 (34.52)	0.832
	Recent travel history	54 (9.84)	27 (10.07)	27 (9.61)	0.855
Duration of symptom before randomize	Mean ± standard deviation (day)	3.06 ± 2.83	2.99 ± 2.63	3.14 ± 3.02	0.559
Comorbidities	At least one Comorbidity *n* (%)	112 (20.4%)	52/211 (19.40%)	60 (21.35%)	0.6790
	Hypertension	43 (7.83)	19 (7.09)	24 (8.54)	0.527
	Diabetes	40 (7.29)	17 (6.34)	23 (8.19)	0.407
	Hypothyroidism	21 (3.83)	9 (3.36)	12 (4.27)	0.577
	Cardiovascular disorders	15 (2.73)	6 (2.24)	9 (3.20)	0.489
	Asthma	13 (2.37)	5 (1.87)	8 (2.85)	0.450
	G6PD deficiency	5 (0.91)	0 (0.0)	5 (1.78)	0.062
	Malignancy	3 (0.55)	2 (0.75)	1 (0.36)	0.616

**TABLE 5:** Symptoms of participants in the first visit of outpatients.

Symptoms	Total (*n* = 549)	Ivermectin (*n* = 268)	Placebo (*n* = 281)	*P-value*
Fever	289 (52.64)	145 (54.10)	144 (51.25)	0.502
Body pain	254 (46.27)	130 (48.51)	124 (44.13)	0.304
Cough	249 (45.36)	130 (48.51)	119 (42.35)	0.147
Headache	209 (38.07)	108 (40.30)	101 (35.94)	0.293
Sore throat	200 (36.43)	103 (38.43)	97 (34.55)	0.341
Chills	171 (31.15)	79 (29.48)	92 (32.74)	0.409
Anorexia	129 (23.50)	59 (22.01)	70 (24.91)	0.424
Weakness	85 (15.48)	33 (12.31)	52 (18.51)	0.059
Anosmia	73 (13.30)	38 (14.18)	32 (12.46)	0.552
Nausea	63 (11.48)	32 (11.94)	31 (11.03)	0.739
Arthralgia	48 (8.74)	24 (8.96)	24 (8.54)	0.864
Diarrhea	48 (8.74)	20 (7.46)	28 (9.96)	0.300
Abdominal pain	47 (8.56)	20 (7.46)	27 (9.61)	0.369
Vertigo	47 (8.56)	23 (8.58)	24 (8.54)	0.986
Ageusia	46 (8.38)	22 (8.21)	24 (8.54)	0.888
Vomiting	35 (6.38)	14 (5.22)	21 (7.47)	0.281
Dyspnea	34 (6.19)	17 (6.34)	17 (6.05)	0.887
Insomnia	34 (6.19)	17 (6.34)	17 (6.05)	0.887
Conjunctivitis	34 (6.19)	17 (6.34)	17 (6.05)	0.887
Tachypnea	15 (2.73)	10 (3.73)	5 (1.78)	0.161
Hypotension	12 (2.19)	4 (1.49)	8 (2.85)	0.278
Wheezing	7 (1.28)	3 (1.12)	4 (1.42)	1
Arthritis	4 (0.73)	3 (1.12)	1 (0.36)	0.362
Cheilitis	4 (0.73)	2 (0.75)	2 (0.71)	1
Skin rash	3 (0.55)	1 (0.37)	2 (0.71)	1

**TABLE 6:** List of concomitant medications, vitamins and minerals supplements prescribed for outpatients.

Medication		Total (*n* = 549)	Ivermectin (*n* = 268)	Placebo (*n* = 281)	*P-value*
Antibiotics	Azithromycin	147 (28.32)	72 (27.80)	75 (28.85)	0.791
	Doxycycline	98 (18.88)	44 (16.99)	54 (20.77)	0.271
	Cefixime	21 (4.03)	13 (5.00)	8 (3.07)	0.262
	Amoxicillin/clavulanic acid	10 (1.93)	5 (1.93)	5 (1.92)	0.995
	Levofloxacin	6 (1.16)	5 (1.93)	1 (0.38)	0.122
	Ciprofloxacin	3 (0.58)	2 (0.77)	1 (0.38)	0.624
	Ampicillin	3 (0.58)	1 (0.39)	2 (0.77)	1
Non-steroidal anti-inflammatory drug	Naproxen	136 (32.46)	60 (29.85)	76 (34.86)	0.274
Other drugs	Famotidine	163 (38.90)	84 (41.79)	79 (36.24)	0.224
Vitamins and minerals supplements	VIT C	210 (50.12)	98 (48.76)	112 (51.38)	0.592
	VIT D	161 (38.42)	69 (34.33)	92 (42.20)	0.098
	Zink	240 (57.28)	116 (57.71)	124 (56.88)	0.864

### Primary Outcomes in Outpatients

**FIGURE 4:**
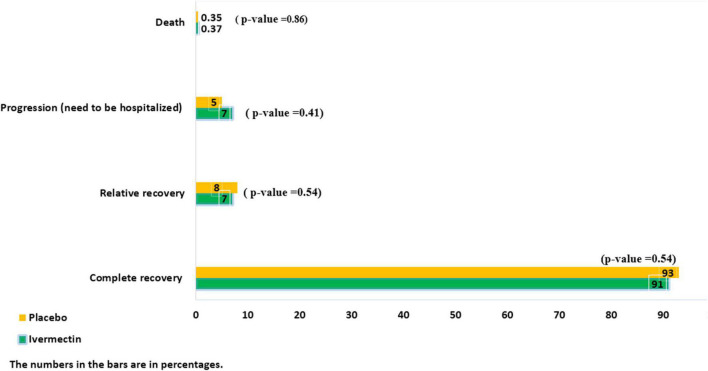
The main outcomes in outpatients with COVID-19.

The mean duration of symptoms in outpatients assigned to ivermectin versus placebo is shown in Table 7. The mean duration of fever was significantly shorter (2.02 ± 0.11 days) in the ivermectin group versus (2.41 ± 0.13 days) placebo group with *p* value = 0.020. Additionally, the mean duration of weakness in the ivermectin group (2.78 ± 0.26 days) was significantly shorter than the placebo group (3.87 ± 0.27 days) with *p* value = 0.002. On the day seventh of treatment, fever (*p*-value = 0.040), cough (*p*-value = 0.019), and weakness (*p*-value = 0.002) were significantly higher in the placebo group compared to the ivermectin group. No significant difference was observed for other symptoms in participants including tachypnea (0.37% of patients in ivermectin versus 0.71% in placebo groups; RR 0.52 [95% CI, 0.5–5.80]; *p*-value = 1; Table 8). Although complete recovery was observed to be higher in the placebo group (93%) compared to the ivermectin group (91%), the differences were not significant between the two groups (RR, 0.98 [95% CI, 0.93–1.04]; *p*-value = 0.54). Relative recovery was seen in 8% of patients in placebo group compared to 7% in the ivermectin group (RR, 1.23 [95% CI, 0.64–2.34]; *p*-value = 0.54). Among all outpatients, 7% in ivermectin group and 5% in placebo group needed to be hospitalized (RR, 1.36 [95% CI, 0.65–2.84]; *p*-value = 0.41) (Figure 4). The result of RT-PCR on day five after treatment was negative for 26% of patients in the ivermectin group versus 32% in the placebo group (RR, 0.81 [95% CI, 0.60–1.09]; *p*-value = 0.16).

**TABLE 7:** Mean duration of symptoms in outpatients.

Duration of symptoms	Group	Mean ± standard error (day)	CI 95%	*P-value* *
Chills	Ivermectin	2.03 ± 0.13	(1.77, 2.28)	0.655
	Placebo	2.17 ± 0.15	(1.87, 2.48)	
Sore throat	Ivermectin	3.10 ± 0.19	(2.73, 3.48)	0.393
	Placebo	3.33 ± 0.19	(2.96, 3.71)	
Cough	Ivermectin	3.87 ± 0.18	(3.51, 4.23)	0.092
	Placebo	4.46 ± 0.18	(4.11, 4.82)	
Shortness breath	Ivermectin	3.67 ± 0.39	(2.89, 4.44)	0.522
	Placebo	3.64 ± 0.36	(2.93, 4.36)	
Anorexia	Ivermectin	4.23 ± 0.29	(3.66, 4.80)	0.688
	Placebo	4.13 ± 0.26	(3.63, 4.63)	
Fever	Ivermectin	2.02 ± 0.11	(1.80, 2.25)	**0.020**
	Placebo	2.41 ± 0.13	(2.16, 2.66)	
Abdominal pain	Ivermectin	2.50 ± 0.29	(1.94, 3.06)	0.749
	Placebo	2.39 ± 0.27	(1.86, 2.91)	
Vertigo	Ivermectin	2.78 ± 0.33	(2.13, 3.44)	0.370
	Placebo	2.45 ± 0.30	(1.87, 3.03)	
Insomnia	Ivermectin	2.48 ± 0.44	(1.62, 3.35)	0.964
	Placebo	2.71 ± 0.38	(1.96, 3.47)	
Arthralgia	Ivermectin	3.20 ± 0.41	(2.40, 4.00)	0.471
	Placebo	3.73 ± 0.43	(2.89, 4.57)	
Headache	Ivermectin	2.58 ± 0.17	(2.24, 2.92)	0.188
	Placebo	2.89 ± 0.19	(2.50, 3.27)	
Nausea	Ivermectin	2.46 ± 0.29	(1.88 ± 3.03)	0.434
	Placebo	2.78 ± 0.29	(2.20 ± 3.35)	
Vomiting	Ivermectin	2.09 ± 0.37	(1.36, 2.82)	0.888
	Placebo	2.09 ± 0.21	(1.67, 2.50)	
Diarrhea	Ivermectin	1.97 ± 0.23	(1.15, 2.43)	0.213
	Placebo	2.37 ± 0.22	(1.95, 2.79)	
Body pain	Ivermectin	3.08 ± 0.18	(2.73, 3.43)	0.212
	Placebo	3.42 ± 0.19	(3.05, 3.78)	
Conjunctivitis	Ivermectin	2.09 ± 0.29	(1.52, 2.66)	0.217
	Placebo	2.80 ± 0.30	(2.22, 3.38)	
Tachypnea	Ivermectin	2.50 ± 0.51	(1.51, 3.49)	0.815
	Placebo	3 ± 0.92	(1.20, 4.79)	
Wheezing	Ivermectin	2.44 ± 0.51	(1.44, 3.45)	0.291
	Placebo	3.25 ± 0.72	(1.84, 4.66)	
Hypotension	Ivermectin	2.20 ± 0.46	(1.29, 3.11)	0.547
	Placebo	3.25 ± 0.63	(2.02, 4.48)	
Ansomnia	Ivermectin	4.02 ± 0.28	(3.48, 4.57)	0.197
	Placebo	4.82 ± 0.26	(4.32, 5.32)	
Ageusia	Ivermectin	3.91 ± 0.37	(3.18, 4.64)	0.065
	Placebo	5.32 ± 0.29	(4.75, 5.90)	
Weakness	Ivermectin	2.78 ± 0.26	(2.26, 3.29)	**0.002**
	Placebo	3.87 ± 0.27	(3.35, 4.39)	

P-value < 0.05 showed by boldface (Kaplan–Meier method*).

**TABLE 8:** Symptoms of outpatients on the seventh day of following.

Symptoms	Total (*n* = 549)	Ivermectin (*n* = 268)	Placebo (*n* = 281)	RR	95% CI	*P-value*
Cough	95 (17.30)	36 (13.43)	59 (21.00)	0.58	(0.37, 0.92)	**0.019**
Anosmia	53 (9.65)	25 (9.33)	28 (9.96)	0.93	(0.53, 1.64)	0.801
Anorexia	44 (8.01)	20 (7.46)	24 (8.54)	0.86	(0.46, 1.60)	0.642
Weakness	42 (7.65)	11 (4.10)	31 (11.03)	0.34	(0.17, 0.70)	**0.002**
Ageusia	38 (6.92)	16 (5.97)	22 (7.83)	0.75	(0.38, 1.46)	0.391
Body pain	36 (6.56)	15 (5.60)	21 (7.47)	0.73	(0.37, 1.46)	0.375
Sore throat	26 (4.74)	10 (3.73)	16 (5.69)	0.64	(0.29, 1.44)	0.279
Dyspnea	26 (4.74)	13 (4.85)	13 (4.63)	1.05	(0.48, 2.31)	0.902
Headache	21 (3.83)	10 (3.73)	11 (3.91)	0.95	(0.40, 2.28)	0.911
Nausea	14 (2.55)	4 (1.49)	10 (3.56)	0.41	(0.13, 1.32)	0.125
Fever	11 (2.00)	2 (0.75)	9 (3.20)	0.23	(0.05, 1.06)	**0.040**
Arthralgia	11 (2.00)	4 (1.49)	7 (2.49)	0.59	(0.17, 2.05)	0.404
Diarrhea	6 (1.09)	2 (0.75)	4 (1.42)	0.52	(0.09, 2.87)	0.686
Chills	5 (0.91)	1 (0.37)	4 (1.42)	0.26	(0.03, 2.33)	0.373
Abdominal pain	5 (0.91)	2 (0.75)	3 (1.07)	0.70	(0.12, 4.20)	1
Vomiting	5 (0.91)	2 (0.75)	3 (1.07)	0.70	(0.12, 4.20)	1
Wheezing	5 (0.91)	2 (0.75)	3 (1.07)	0.70	(0.12, 4.20)	1
Vertigo	4 (0.73)	3 (1.12)	1 (0.36)	3.17	(0.33, 30.66)	0.362
Insomnia	4 (0.73)	2 (0.75)	2 (0.71)	1.05	(0.15, 7.50)	1
Hypotension	4 (0.73)	1 (0.37)	3 (1.37)	0.35	(0.4, 3.36)	0.624
Tachypnea	3 (0.55)	1 (0.37)	2 (0.71)	0.52	(0.5, 5.80)	1
Cheilitis	2 (0.36)	1 (0.37)	1 (0.36)	1.05	(0.06, 16.85)	1
Conjunctivitis	2 (0.36)	1 (0.37)	1 (0.36)	1.05	(0.06, 16.85)	1

*P-value < 0.05 showed by boldface.*

### Secondary Outcomes in Outpatients

Few patients needed to be admitted in the ICU (0.5% in the ivermectin group and 0.4% in the placebo group (RR, 1.09 [95% CI, 0.07–17.32) *p*-value = 0.95), and there was no significant difference between the two treatment groups. Adverse side effects, including itching and skin rash, were observed in only one patient in the ivermectin group from the second to the fifth day of treatment. Moreover, death was observed in one patient in both groups (Figure 4).

## Discussion

Ivermectin is a low-cost established drug with clinical benefits and minimal safety concerns, which has been shown to inhibit SARSCoV-2 *in vitro* in studies (20, 26). Ivermectin has rapid oral absorption, with high lipid solubility is widely circulated in the body, metabolized in the liver, and excreted in feces (29). The adequate concentration of ivermectin inhibiting SARS-CoV-2 in the *in vitro* experiment is higher than the approved dose of ivermectin concentration in plasma and the lungs of humans (30). However, a meta-analysis demonstrated that the administration of a standard FDA-approved dose shows a positive clinical response in COVID-19 patients (26).

We conducted two multicenter randomized, double-blind, placebo-controlled clinical trials evaluating the effectiveness of ivermectin on inpatients and outpatients with COVID-19 in Iran.

It is noted that the patients’ characterizations, including age, gender, duration of COVID-19 symptoms before randomization, the severity of disease, and comorbidities, were matched in both groups (ivermectin and placebo) in these clinical trials. In our clinical trials, although ivermectin was well-tolerated in mild to severe COVID-19 patients, there were no significant clinical benefits demonstrated for treating COVID-19 with a 0.4 mg/kg/day dose over a duration of 3 days. We observed that not only was there no significant potential effectiveness of ivermectin on clinical improvement, resolution of symptoms, reduced admission in ICU, need for invasive ventilation, and death in inpatients; no evidence was found to support the prescription of ivermectin on recovery, reduced hospitalization and increase negative RT-PCR assay for SARS-CoV-2 5 days after treatment in outpatients. To the best of our knowledge, this is the first comprehensive randomized, double-blind, placebo-controlled, study where both inpatients and outpatients were evaluated. Despite our previous more favorable results from a multicenter, randomized clinical trial in 69 COVID-19 patients at the beginning of the pandemic which noted the effectiveness of ivermectin in recovery and decreasing duration of hospital stay, the current results of this extensive study on 609 admitted patients with moderate to severe form of COVID-19 and 549 outpatients with a mild form of COVID-19, did not show adequate support for the effectiveness of this drug (28). Although several studies and some meta-analyses appear to confirm the efficacy of ivermectin in reducing the symptoms or length of hospital stay and mortality due to COVID-19, a limitation of these conclusions is the small size and quality of primary studies (26, 28, 31, 32). Notwithstanding these previous reports, we found ivermectin (37%) compared with placebo (28%) may make some difference in the complete recovery of patients on discharge day, but the length of hospital stay in the placebo group was significantly shorter than ivermectin arm. In a meta-analysis by Hill et al., which analyzed ivermectin in 23 randomized clinical trials, Ivermectin did not demonstrate a statistically significant result on hospitalizations. Although, it showed a borderline impact on the duration of hospital admission compared to SOC (30). Also, Ozer et al., in a prospective observational cohort study, did not find significant differences between ivermectin and control groups regarding the length of hospital stay, ICU admission, intubation rate, and in-hospital mortality (33). Delays in discharging patients to other facilities such as rehabilitation centers, co-infections such as bacterial infection might be the reason for more extended hospital stay other than treatment for COVID-19. In our study, in outpatients with a mild form of COVID-19, ivermectin showed significant effectiveness in reducing the mean duration of symptoms such as fever and weakness. Also, on the seventh day after treatment, symptoms such as fever, cough, and weakness were significantly lower in the ivermectin group compared to the placebo group. In contrast to our finding, López-Medina et al., found in a randomized clinical trial among 398 patients, the duration of symptoms was not significantly different (10 days in the ivermectin group versus 12 days in the placebo group), and they reported that Ivermectin is not beneficial to symptom resolution in mild COVID-19 patients (34). The differences in our findings with López-Medina et al. might be explained by the longer-term following of the symptom, which they did until day 21, and in contrast, we followed patients until day seven after the first visit. In the current study, the rate of complete and relative recovery, need for hospitalization, and negativity of RT-PCR after treatment showed the non-effectiveness of ivermectin in the mild form of COVID-19; therefore, the ability of ivermectin to deter the progression of mild form to moderate or severe form of COVID-19 failed in this large clinical trial. In line with our results in a systematic review by Roman et al., ivermectin in comparing SOC or placebo did not reduce outcomes such as mortality rate, length of hospital stay, adverse events, and SARS-CoV-2 clearance in respiratory samples (35). On the other hand, Krolewiecki et al. assessed antiviral activity and safety of a 5-day regimen of high dose ivermectin, comparing the control group in 45 patients with COVID-19. The findings support the hypothesis that ivermectin has a concentration-dependent antiviral activity against SARS-CoV-2 (36).

At standard doses of 0.2–0.4 mg/kg for 1–2 days, ivermectin has a good safety profile (30). After a standard oral dose administration in healthy humans, ivermectin reaches peak plasma levels at less than 5 h (37). The half-life of ivermectin in blood and lungs is different, and various doses of ivermectin may show different effects. Although ivermectin concentrations in lung tissue cannot be measured in humans, it is estimated to accumulate in lung tissues 2.67 times higher than plasma. However, whether this concentration can produce pulmonary antiviral activity is unclear. Heretofore several clinical trials investigated ivermectin as a single dose (0.2–0.4 mg/kg) with sample sizes of 62–363 patients (21, 28, 38–40)or multiday dosing of 2 up to 5 days with sample size 45–500 patients (34, 36, 40). Ivermectin which was widely prescribed as a potential treatment for COVID-19 at the beginning of the pandemic, showed uncertain clinical benefit in many clinical trials, including our study.

The potential toxic effect of ivermectin is neurotoxicity, including severe episodes of confusion, ataxia, seizures (41). Moreover, nausea, vomiting, diarrhea, hypotension, itching, and hives are other adverse effects of ivermectin which can lead to a mistake in distinguishing between the symptoms of COVID-19 and the side effects of ivermectin. Subsequently, it may be underestimating the effect of ivermectin in the resolution of COVID-19 symptoms.

Although we did not observe the serious adverse effect following administration of ivermectin in both clinical trials, the increased visitations to poison control centers about ivermectin toxicity compared with pre-pandemic rates have been reported. Oral use of ivermectin in outpatients requires precise and well-defined instructions and education to avoid any overdose that could lead to poisoning (35, 42, 43). The current study had some drawbacks which might influence the conclusions. First, after the allocation of ivermectin or placebo, a significant number of patients declined to be participants. Second, we did not perform long-term follow-up of patients after discharge to evaluate the resolution of symptoms. Third, the facilities of the hospitals varied in terms of the number of active beds and ICU. Therefore, it affected the length of stay or hospitalization of patients. On the other hand, this study had some limitations. Due to the lack of facilities, we didn’t conduct the virological assessment to evaluate the role of ivermectin in viral clearance in hospitalized patients and the measurement of ivermectin plasma levels to obtain insights into the antiviral effect of ivermectin in COVID-19 patients. Also, ivermectin may be going to be effective if it is given at the earliest possible time that clinical symptoms appear whiles the mean duration of symptoms before randomization was 7.36 ± 3.43 days in the ivermectin group and 6.98 ± 3.63 days in the placebo group.

Nevertheless, despite some drawbacks and limitations, important conclusions may be drawn from these clinical trials.

## Conclusion

Ivermectin, compared with placebo, did not improve clinical recovery, reduce admission in ICU, reduce the need for invasive ventilation, and death in inpatients. For outpatients, ivermectin did not improve recovery, reduce hospitalization, or increase negative RT-PCR assay for SARS-CoV-2 after treatment. Our findings do not support the use of ivermectin for treatment of mild to severe form of COVID-19.

## Data Availability Statement

The original contributions presented in this study are included in the article/Supplementary Material, further inquiries can be directed to the corresponding author.

## Ethics Statement

The studies involving human participants were reviewed and approved by the Ethics Committee of Mazandaran University of Medical Sciences (IR.MAZUMS.REC.1399.915 and IR.MAZUMS.REC.1399.869) and by the Iranian Registry of Clinical Trials identifier (IRCT20111224008507N5 and IRCT20111224008507N4). Written informed consent to participate in this study was provided by the participants or their legal guardian/next of kin.

## Author Contributions

MR contributed to the conception or design of the work. MR, FA, AHi, LE, and MMi contributed to the drafting and statistical analysis of the manuscript. All authors contributed toward the acquisition, analysis, or interpretation of data, critical revision of the manuscript, review and approval of the final version of the manuscript.

## Conflict of Interest

The authors declare that the research was conducted in the absence of any commercial or financial relationships that could be construed as a potential conflict of interest.

## Publisher’s Note

All claims expressed in this article are solely those of the authors and do not necessarily represent those of their affiliated organizations, or those of the publisher, the editors and the reviewers. Any product that may be evaluated in this article, or claim that may be made by its manufacturer, is not guaranteed or endorsed by the publisher.
