# Supplementary material for: Non-effectiveness of Ivermectin on Inpatients and Outpatients With COVID-19; Results of Two Randomized, Double-Blinded, Placebo-Controlled Clinical Trials
Source: Front Med (Lausanne). 2022 Jun 16;9:919708. doi: 10.3389/fmed.2022.919708 (PMC9244711; doi:10.3389/fmed.2022.919708)
Supplement: Supplementary file 1 [file Data_Sheet_1.PDF]

Clinical Trial Protocol
Iranian Registry of Clinical Trials

15 Mar 2022

Double-blind placebo-controlled clinical trial of evaluating the effectiveness of Ivermectin in treatment of patients admitted with COVID-19 in 2021

Protocol summary

Study aim

Determining the effect of ivermectin on RT-PCR test, clinical improvement, mortality and duration of hospitalization in patients admitted with COVID-19

Design

Clinical trial with control group, parallel groups, double-blind, randomized, phase 3 on 1000 patients. Patients will be divided into two groups by a simple randomization method with a table of random numbers. The control group will receive standard and placebo treatment and the intervention group will receive Ivermectin for three days in addition to the standard treatment.

Settings and conduct

COVID-19 positive rapid test or RT-PCR patients admitted to Buali and Imam hospitals of Sari, Razi hospital of Qaemshahr, Imam Hossein hospital of Neka and Imam hospital of Fereidoonkenar will be divided into two groups of intervention and control. The present study will be double-blind so that patients and physicians will be unaware of how the intervention and control groups assigned.

Participants/Inclusion and exclusion criteria

Inclusion criteria: Patients with COVID-19 positive rapid test or RT-PCR; aged >5 years; weight more than 15 kg and without liver and lung disease and acquired immunodeficiency and pregnancy lactation and without treatment with antiviral drugs before and during the study are included.

Intervention groups

Intervention group: Iranian standard treatment protocol for COVID-19 in addition to 6mg Ivermectin tablet made by Alborz Daru Company of Iran for 3 days. Control group: In the control group, placebo tablets made by Alborz Daru Company of Iran with the same shape, color and weight based dose of ivermectin will be used for three days.

Main outcome variables

Clinical improvement, Duration of hospital stay, Rate of Mortality

General information

Reason for update

Increase in sample size and pharmaceutic company

Acronym

IRCT registration information

IRCT registration number: **IRCT20111224008507N5**
Registration date: **2021-02-22, 1399/12/04**
Registration timing: **registered_while_recruiting**

Last update: **2021-03-04, 1399/12/14**
Update count: **1**

Registration date

2021-02-22, 1399/12/04

Registrant information

Name

Mohammadsadegh Rezai

Name of organization / entity

Mazandaran University of Medical Sciences

Country

Iran (Islamic Republic of)

Phone

+98 15 1325 7230

Email address

rezai@mazums.ac.ir

Recruitment status

Recruitment complete

Funding source

Expected recruitment start date

2021-02-19, 1399/12/01

Expected recruitment end date

2021-08-22, 1400/05/31

Actual recruitment start date

empty
Actual recruitment end date
empty
Trial completion date
empty

Scientific title
Double-blind placebo-controlled clinical trial of evaluating the effectiveness of Ivermectin in treatment of patients admitted with COVID-19 in 2021

Public title
Evaluation of the effect of Ivermectin in treatment of patients admitted with COVID-19
Purpose
Treatment
Inclusion/Exclusion criteria
Inclusion criteria:
Patients with positive coronavirus rapid test or RT-PCR Age>5 years Weight >15 kg No treatment with antiviral drugs before and during the study Informed consent for participation
Exclusion criteria:
Underlying liver and kidney disease Patients with acquired immunodeficiency Pregnancy and lactation
Age
From **5 years** old
Gender
Both

Phase
3
Groups that have been masked

- Participant
- Investigator

Sample size
Target sample size: **1000**
Randomization (investigator's opinion)
Randomized
Randomization description
First, using the Random number generation plugin in excel software, a table of random numbers from 1 to 1000 is prepared in a non-sequential and scattered manner, and the numbers are assigned to two intervention and control groups of 500 cases. The randomization process is performed by the methodology consultant and clinical researchers are not aware of the randomization process and will only be provided with random codes from 1 to 1000.
Blinding (investigator's opinion)
Double blinded
Blinding description
After selecting the samples, none of the participants will be aware of randomization and allocation to groups. Physicians will be given a table of pre-coded numbered numbers and patients will be entered into the study in order of table numbers. Therefore, the present study will be double-blind. Ivermectin and placebo tablets will be in the same shape, color and size and will be delivered to the patient/parents in a package.
Placebo
Used
Assignment
Parallel
Other design features

Secondary Ids

empty

Ethics committees

1

Ethics committee
Name of ethics committee
Ethics committee of Mazandaran University of Medical Sciences
Street address
Vice chancellor for Research, Moallem square, Sari
City
Sari
Province
Mazandaran
Postal code
4712855689
Approval date
2021-01-27, 1399/11/08
Ethics committee reference number
IR.MAZUMS.REC.1399.915

Health conditions studied

1

Description of health condition studied
COVID-19 infection
ICD-10 code
U07.1
ICD-10 code description
COVID-19, virus identified

Primary outcomes

1

Description
Reduction in persistent cough
Timepoint
Daily until improvement
Method of measurement
Question from the patient

2

Description
Negative RT-PCR result
Timepoint
6 days after the intervention
Method of measurement
RT-PCR

3

Description
The main complaints recovery time
Timepoint
Daily until symptoms resolve
Method of measurement
Checklist containing patient complaints

4

Description
Mortality
Timepoint
Daily
Method of measurement
Record in checklist

5

Description
Drug side effect (Wheezing, itching, skin rash, edema, and hypotension)
Timepoint
Daily
Method of measurement
Question from the patient

6

Description
Reduction in tachypnea
Timepoint
Daily
Method of measurement
Medical record

7

Description
Oxigen saturation >94%
Timepoint
Daily
Method of measurement
Medical record

Secondary outcomes

empty

Intervention groups

1

Description
Intervention group: The intervention group will use Iranian standard treatment protocol for COVID-19 in addition to Ivermectin 3 mg oral tablet at a dose of 0.4 mg/kg manufactured by Alborz Daru of Iran for 3 days as follows: weight 15-24, 6 mg; Weight 35-25, 12 mg; Weight 50-36, 18 mg; Weight 80-5, 24 mg and weight over 80, 0.4 mg/kg
Category
Treatment - Drugs

2

Description
Control group: In the control group, placebo tablets made by Alborz Daru of Iran with the same shape, color and weight based dose of ivermectin will be used for three days.
Category
Placebo

Recruitment centers

1

Recruitment center
Name of recruitment center
Imam and Buali hospitals of Sari, Razi hopspital of Qaemshahr, Imam Hossein hospital of Neka and Ima
Full name of responsible person
Dr Mohammad Sadegh Rezai
Street address
Bouali Hospital, Pasdaran boulevard, Sari
City
Sari
Province
Mazandaran
Postal code
4815838477
Phone
+98 11 3334 2334
Email
drmsrezaii@yahoo.com

Sponsors / Funding sources

1

Sponsor
Name of organization / entity
Mazandaran University of Medical Sciences
Full name of responsible person
Dr. Majid Saeidi
Street address
Vice chancellor for Research, Moallem square, Sari
City
Sari
Province
Mazandaran
Postal code
4712855689
Phone
+98 11 3334 2334
Email
msaidi@mazums.ac.ir
Grant name
Grant code / Reference number
Is the source of funding the same sponsor organization/entity?
Yes
Title of funding source

Mazandaran University of Medical Sciences

Proportion provided by this source
100

Public or private sector
Public

Domestic or foreign origin
Domestic

Category of foreign source of funding
empty

Country of origin

Type of organization providing the funding
Academic

Person responsible for general inquiries

Contact

Name of organization / entity
Mazandaran University of Medical Sciences

Full name of responsible person
Dr Mohammad Sadegh Rezai

Position
Professor

Latest degree
Subspecialist

Other areas of specialty/work
Pediatrics

Street address
Boali Hospital, Pasdaran Blv., Sari

City
Sari

Province
Mazandaran

Postal code
4815838477

Phone
+98 11 3334 2334

Fax
+98 11 3334 2334

Email
drmsrezaii@yahoo.com

Web page address

Person responsible for scientific inquiries

Contact

Name of organization / entity
Mazandaran University of Medical Sciences

Full name of responsible person
Dr Mohammad Sadegh Rezai

Position
Professor

Latest degree
Subspecialist

Other areas of specialty/work
Pediatrics

Street address
Bouali Hospital, Pasdaran Boulevard, Sari

City
Sari

Province
Mazandaran

Postal code
4815838477

Phone
+98 11 3334 2334

Email
drmsrezaii@yahoo.com

Person responsible for updating data

Contact

Name of organization / entity
Mazandaran University of Medical Sciences

Full name of responsible person
Fatemeh Hosseinzadeh

Position
Research Expert

Latest degree
Master

Other areas of specialty/work
Midwifery

Street address
Bouali Hospital, Pasdaran Boulevard, Sari

City
Sari

Province
Mazandaran

Postal code
4815838477

Phone
+98 11 3334 2334

Email
fatima.hzade@gmail.com

Sharing plan

Deidentified Individual Participant Data Set (IPD)
Yes - There is a plan to make this available

Study Protocol
Yes - There is a plan to make this available

Statistical Analysis Plan
Not applicable

Informed Consent Form
No - There is not a plan to make this available

Clinical Study Report
Yes - There is a plan to make this available

Analytic Code
Not applicable

Data Dictionary
Not applicable

Title and more details about the data/document
Part of the data is accessible

When the data will become available and for how long
Starting in January 2022

To whom data/document is available
Physicians

Under which criteria data/document could be used
Systematic review articles

From where data/document is obtainable
Contact Dr. Mohammad Sadegh Rezai. E-mail: drmsrezaii@yahoo.com

What processes are involved for a request to access

data/document After contact, information is sent within a few days

Comments
