# Supplementary material for: Non-effectiveness of Ivermectin on Inpatients and Outpatients With COVID-19; Results of Two Randomized, Double-Blinded, Placebo-Controlled Clinical Trials
Source: Front Med (Lausanne). 2022 Jun 16;9:919708. doi: 10.3389/fmed.2022.919708 (PMC9244711; doi:10.3389/fmed.2022.919708)
Supplement: Supplementary file 2 [file Data_Sheet_2.PDF]

Clinical Trial Protocol
Iranian Registry of Clinical Trials

15 Mar 2022

Double-blind placebo-controlled clinical trial of evaluating the effectiveness of Ivermectin in treatment of outpatients with COVID-19 in 2021

Protocol summary

Study aim

Determining the effect of ivermectin on PCR test, clinical improvement, percentage and duration of hospitalization in outpatients with COVID-19

Design

Clinical trial with control group, parallel groups, double-blind, randomized, phase 3 on 1000 patients. Patients are divided into two groups by a simple randomization method with a table of random numbers. The control group will receive standard and placebo treatment and the intervention group will receive Ivermectin for three days in addition to the standard treatment.

Settings and conduct

COVID-19 positive rapid test patients referred to family physician and infectious disease specialist are divided into two groups of intervention and control. The present study is double-blind so that patients and physicians will be unaware of how the intervention and control groups are assigned.

Participants/Inclusion and exclusion criteria

Patients with COVID-19 positive rapid test or RT-PCR age >5 years and weight >15 kg are included in the study

Intervention groups

The intervention group will use Iranian standard treatment protocol for COVID-19 in addition to Ivermectin 6 mg oral tablet at a dose of 0.4 mg/kg manufactured by Alborz Daru Co of Iran for 3 days as follows: weight 15-24, 6 mg; Weight 35-25, 12 mg; Weight 50-36, 18 mg; Weight 80-5, 24 mg and weight over 80, 0.4 mg/kg

Main outcome variables

The primary outpoints are clinical improvement and negative PCR result after 6 days. Clinical improvement is defined as reduction in persistent cough (more than one hour of excessive coughing, or 3 periods of coughing in 24 hours that disrupts daily life and ability to work) and tachypnea and O2 saturation above 94%.

General information

Reason for update

Increase in sample size

Acronym

IRCT registration information

IRCT registration number: **IRCT20111224008507N4**
Registration date: **2021-01-31, 1399/11/12**
Registration timing: **prospective**

Last update: **2021-03-06, 1399/12/16**
Update count: **1**

Registration date

2021-01-31, 1399/11/12

Registrant information

Name
Mohammadsadegh Rezai
Name of organization / entity
Mazandaran University of Medical Sciences
Country
Iran (Islamic Republic of)
Phone
+98 15 1325 7230
Email address
rezai@mazums.ac.ir

Recruitment status

Recruitment complete

Funding source

Expected recruitment start date

2021-02-19, 1399/12/01

Expected recruitment end date

2021-08-22, 1400/05/31

Actual recruitment start date

empty

Actual recruitment end date

empty

Trial completion date
empty

Scientific title
Double-blind placebo-controlled clinical trial of evaluating the effectiveness of Ivermectin in treatment of outpatients with COVID-19 in 2021

Public title
Evaluation of the effect of Ivermectin in treatment of outpatients with COVID-19

Purpose
Treatment

Inclusion/Exclusion criteria
Inclusion criteria:
Patients with positive coronavirus rapid test or RT-PCR positive No need for hospitalization Weight >15 kg Age >5 years No treatment with antiviral drugs before and during the study Informed consent for inclusion
Exclusion criteria:
Underlying liver and kidney disease Patients with acquired immunodeficiency Pregnancy and lactation

Age
From **5 years** old

Gender
Both

Phase
3

Groups that have been masked

- Participant
- Care provider

Sample size
Target sample size: **1000**

Randomization (investigator's opinion)
Randomized

Randomization description
Participants will be randomly assigned to two groups of intervention and control with 500 members using block randomization with block sizes of 4. Randomization will be done using the software randomization option in Excel. The randomization process is performed by the study methodology consultant and clinical researchers are not aware of the randomization process.

Blinding (investigator's opinion)
Double blinded

Blinding description
After selecting the samples, none of the participant will be aware of randomization and allocation to groups. Physicians are given a table of pre-coded numbered numbers and patients are entered into the study in order of table numbers. Therefore, the present study is double-blind. Ivermectin and placebo tablets are the same shape, color and size and are delivered to the patient/parents in a package.

Placebo
Used

Assignment
Parallel

Other design features

Secondary Ids

empty

Ethics committees

1

Ethics committee
Name of ethics committee
Ethics committee of Mazandaran University of Medical Sciences
Street address
Vice chancellor for Research, Moallem square, Sari
City
Sari
Province
Mazandaran
Postal code
4815838477

Approval date
2020-12-30, 1399/10/10

Ethics committee reference number
IR.MAZUMS.REC.1399.869

Health conditions studied

1

Description of health condition studied
COVID-19 infection

ICD-10 code
B34.2

ICD-10 code description
Coronavirus infection, unspecified

Primary outcomes

1

Description
Clinical improvement

Timepoint
Daily until improvement

Method of measurement
Clinical improvement is defined as reduction in persistent cough (more than one hour of excessive coughing, or 3 periods of coughing in 24 hours that disrupts daily life and ability to work) and tachypnea and O2 saturation above 94%.

2

Description
Negative PCR result

Timepoint
6 days after intervention

Method of measurement
RT-PCR

Secondary outcomes

1

Description
The main complaints recovery time
Timepoint
Daily until symptoms resolve
Method of measurement
Checklist containing patient complaints

2

Description
Need to be hospitalized
Timepoint
Daily until hospitalization or improvement
Method of measurement
Percentage of hospitalization and the interval from the beginning of the intervention to hospitalization

3

Description
Mortality
Timepoint
Daily
Method of measurement
Record in checklist

4

Description
Drug side effect
Timepoint
Daily
Method of measurement
Wheezing, itching, skin rash, edema, and hypotension are assessed daily

Intervention groups

1

Description
Intervention group: The intervention group will use Iranian standard treatment protocol for COVID-19 in addition to Ivermectin 6 mg oral tablet at a dose of 0.4 mg/kg manufactured by Alborz Daru Co, Iran for 3 days as follows: weight 15-24, 6 mg; Weight 35-25, 12 mg; Weight 50-36, 18 mg; Weight 80-5, 24 mg and weight over 80, 0.4 mg/kg
Category
Treatment - Drugs

2

Description
Control group: In the control group, placebo tablets made by Alborz Daru company of Iran with the same shape, color and weight based dose of ivermectin will be used for three days.
Category
Placebo

Recruitment centers

1

Recruitment center
Name of recruitment center
Office of family physicians and infectious disease specialists and pediatric infectious diseases sub
Full name of responsible person
Dr Mohammad Sadegh Rezai
Street address
Bouali Hospital, Pasdaran boulevard, Sari
City
Sari
Province
Mazandaran
Postal code
4815838477
Phone
+98 11 3334 2334
Email
drmsrezaii@yahoo.com

Sponsors / Funding sources

1

Sponsor
Name of organization / entity
Mazandaran University of Medical Sciences
Full name of responsible person
Dr. Majid Saeidi
Street address
Vice chancellor for Research, Moallem square, Sari
City
Sari
Province
Mazandaran
Postal code
4712855689
Phone
+98 11 3334 2334
Email
msaidi@mazums.ac.ir
Grant name
Grant code / Reference number
Is the source of funding the same sponsor organization/entity?
Yes
Title of funding source
Mazandaran University of Medical Sciences
Proportion provided by this source
100
Public or private sector
Public
Domestic or foreign origin
Domestic
Category of foreign source of funding
empty
Country of origin

Type of organization providing the funding
Academic

Person responsible for general inquiries

Contact
Name of organization / entity
Mazandaran University of Medical Sciences
Full name of responsible person
Dr. Mohammad Sadegh Rezai
Position
Professor
Latest degree
Subspecialist
Other areas of specialty/work
Infectious diseases
Street address
Bouali Hospital, Pasdaran boulevard, Sari
City
Sari
Province
Mazandaran
Postal code
4815838477
Phone
+98 11 3334 2334
Email
drmsrezaii@yahoo.com

Person responsible for scientific inquiries

Contact
Name of organization / entity
Mazandaran University of Medical Sciences
Full name of responsible person
Dr. Mohammadsadegh Rezai
Position
Professor
Latest degree
Subspecialist
Other areas of specialty/work
Infectious diseases
Street address
Bouali Hospital, Pasdaran Boulevard, Sari
City
Sari
Province
Mazandaran
Postal code
4815838477
Phone
+98 11 3334 2334
Email
drmsrezaii@yahoo.com

Person responsible for updating data

Contact
Name of organization / entity
Mazandaran University of Medical Sciences
Full name of responsible person
Fatemeh Hosseinzadeh
Position
Officer
Latest degree
Master
Other areas of specialty/work
Psychology
Street address
Bouali Hospital, Pasdaran Boulevard, Sari
City
Sari
Province
Mazandaran
Postal code
4815838477
Phone
+98 11 3334 2334
Email
fatima.hzade@gmail.com

Sharing plan

Deidentified Individual Participant Data Set (IPD)
Yes - There is a plan to make this available
Study Protocol
Yes - There is a plan to make this available
Statistical Analysis Plan
Not applicable
Informed Consent Form
Yes - There is a plan to make this available
Clinical Study Report
Yes - There is a plan to make this available
Analytic Code
No - There is not a plan to make this available
Data Dictionary
No - There is not a plan to make this available
Title and more details about the data/document
Part of the data is accessible
When the data will become available and for how long
Starting in January 2022
To whom data/document is available
Everybody
Under which criteria data/document could be used
Systematic review articles
From where data/document is obtainable
Contact Dr. Mohammad Sadegh Rezai. E-mail: drmsrezaii@yahoo.com
What processes are involved for a request to access data/document
After contact, information is sent within a few days
Comments
